# Comprehensive and quantitative profiling of B vitamins and related compounds in the mammalian liver

**DOI:** 10.1016/j.jchromb.2019.121884

**Published:** 2020-01-01

**Authors:** Juan Xu, Constance E. Clare, Amey H. Brassington, Kevin D. Sinclair, David A. Barrett

**Affiliations:** a Centre for Analytical Bioscience, Division of Advanced Materials and Healthcare Technologies, School of Pharmacy, University of Nottingham, Nottingham NG7 2RD, UK; b School of Bioscience, University of Nottingham, Sutton Bonington, Leicestershire LE12 5RD, UK

**Keywords:** B vitamins, Amines, One-carbon metabolism, HILIC, Mass spectrometry, Sheep liver

## Abstract

A method for the simultaneous quantification of B vitamins and related amines in one-carbon (1C) metabolism would benefit the study of diet and genetic/epigenetic regulation of mammalian development and health. We present a validated method for the simultaneous quantitative analysis of 13 B vitamers and four related 1C-pathway amine intermediates in liver using hydrophilic interaction chromatography (HILIC) coupled to electrospray ionization tandem mass spectrometry. Frozen sheep liver samples (50 mg) were homogenized in cold 50% acetonitrile containing 1% acetic acid with the addition of two isotope labelled internal standards. Hot acid hydrolysis was applied to release the protein-bound forms. The separation of 17 analytes was achieved using a pHILIC column with a total run time of 13 min. Detection was achieved in electrospray positive ionisation mode. Limits of detection for the majority of analytes were within the range of 0.4–3.2 pmol/g. The method was applied to 266 sheep liver samples and revealed that adenosylcobalamin, methylcobalamin, pyridoxic acid, flavin adenine dinucleotide and thiamine were the major forms of the B vitamers present with pyridoxal 5′-phosphate and thiamine pyrophosphate being detected at lower concentrations. Trimethylglycine and methylglycine were the predominant 1C-related amines measured. As anticipated, the B vitamin status of individuals varied considerably, reflecting dietary and genetic variation in our chosen outbred model species. This method offers a simple sample extraction procedure and provides comprehensive coverage of B vitamins coupled with good sensitivity and reliability.

## Highlights

• A validated method for quantification of 13 B vitamins and four related compounds in sheep liver.
• Limits of detection for the majority of analytes were within the range of 0.4–3.2 pmol/g.
• Simple sample extraction procedure with high throughput.
• Successfully applied to profile 1C major forms in 266 sheep liver samples.
• Potential for dietary and genetic studies in metabolic health and epigenetic gene regulation.

## 1 Introduction

**Fig. 1:**
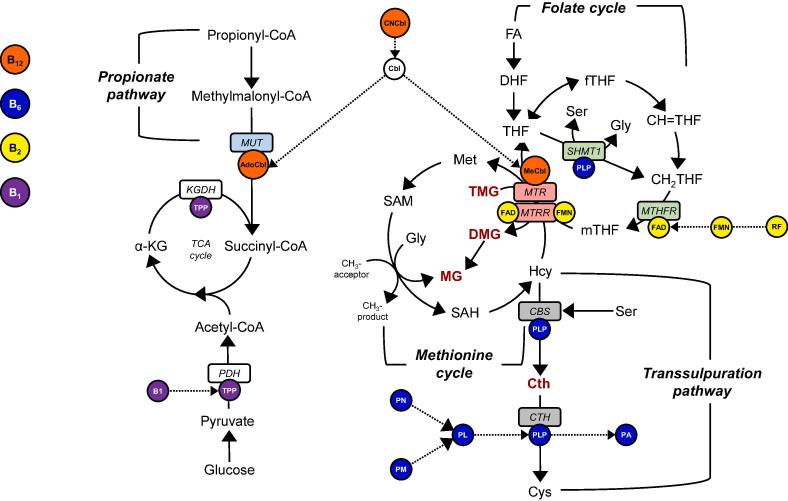
B-vitamins, one carbon metabolism and related pathways. Folate cycle enzymes annotated in green boxes: MTHFR, 5,10-methylenetetrahydrofolate reductase; SHMT1, serine hydroxymethyltransferase. *Methionine cycle enzymes annotated in red boxes*: MTR, methionine synthase; MTRR, methionine synthase reductase. Transsulphuration pathway enzymes annotated in grey boxes: CBS, cystathionine β-synthase; CTH, cystathionine Υ-lyase. *Propionate pathway enzyme annotated in blue box*: MUT, methylmalonyl-CoA mutase. *Tricarboxylic acid cycle enzymes in white boxes*: KGDH, α-ketoglutarate dehydrogenase; PDH, pyruvate dehydrogenase; SHMT1, serine hydroxymethyltransferase. *Vitamin B1 cofactors annotated in purple circles*: B1, thiamine; TPP, thiamine pyrophosphate. Vitamin B2 cofactors annotated in yellow circles: RF, riboflavin; FMN, flavin mononucleotide; FAD, flavin adenine dinucleotide. *Vitamin B6 cofactors annotated in blue circles:* PN, pyridoxine; PM, pyridoxamine; PL, pyridoxal; PLP, pyridoxal 5′-phosphate; PA, 4-pyridoxic acid. *Vitamin B12 cofactors annotated in orange circles*: CNCbl, cyanocobalamin; MeCbl, methylcobalamin; AdoCbl, adenosylcobalamin. *White circle*: Cbl, cobalamin intermediate. *Key 1C-related amines annotated in red text*: Cth, cystathionine; TMG, trimethylglycine; DMG, dimethylglycine; MG, methylglycine. *Folate cycle substrates*: FA, folic acid; DHF, dihydrofolate; THF, tetrahydrofolate; fTHF, 10-formyltetrahydrofolate; CH = THF, 5,10-methenyltetrahydrofolate; CH2THF, 5,10-methylenetetrahydrofolate; mTHF, 5-methyltetrahydrofolate. *Methionine cycle substrates*: Met, methionine, SAM, S-adenosylmethionine; SAH, S-adenosylhomocysteine; Hcy, homocysteine; Gly, glycine; Ser, serine. *Transsulphuration pathway substrate*: Cys, cysteine. *Tricarboxylic acid cycle substrate*: α-KG, α-ketoglutarate. Solid arrows demonstrate flux through metabolic pathways. Dotted arrows demonstrate interconversion and interaction of B-vitamin species with metabolic pathways.

One carbon metabolism (1C) comprises a series of biochemical pathways that provides methyl groups for a wide range of biological processes. The impairment of these pathways has potential to cause serious disruption to DNA synthesis and repair, and epigenetic gene regulation, all of which can have an adverse effect on the health of an organism [1], [2]. One carbon metabolism can be monitored by the quantitative measurement of key intermediates in these pathways either in blood or in a metabolically active tissue such as the liver. The B group of vitamins (Table S1 in Supplementary Information) are co-substrates of various reactions involved in methylation, including homocysteine (Hcy) remethylation, and typically function as either a reserve pool or as a direct precursor for the synthesis of coenzymes involved in 1C metabolism more generally [3] (Fig. 1). Specific 1C-related amines also modulate Hcy status by facilitating remethylation to regenerate methionine or catabolism via the transsulphuration pathway [4], [5]. Quantitative assessments of these 1C intermediates and co-substrates provide valuable insights into dietary-mediated 1C metabolic function.

Active variants of B vitamins are present in biofluids and tissues in free and phosphorylated forms at varying concentrations and are known to be susceptible to degradation by light, heat, oxygen and pH [6]. In addition, the chemical diversity of B vitamins and related compounds results in a wide range of physicochemical properties, which presents a significant analytical challenge to develop a single method for their simultaneous determination.

A number of analytical approaches have been developed for the determination of B vitamins including microbiological assays, immunoassays, and HPLC coupled to electrochemical, ultraviolet or fluorescence detection [7], [8], [9], [10], [11]. However, most studies have focused on individual or a small subset of vitamers without the capacity to analyze a comprehensive set of these variants in one analytical run. Previous LC-MS/MS methods have monitored B vitamins in fruit and vegetables [12], [13]. However, despite good sensitivity, neither of these methods included a comprehensive coverage of the biologically active forms of B12, B2 and B1, and they involved complex and time-consuming sample preparation procedures. Moreover, B vitamins are often extracted by acid and enzymatic hydrolysis and determined by the total content of vitamins while the chemically distinct bioactive forms are not individually measured. Although methods for measuring specific B vitamins in biofluids exist [14], [15], [16], [17], none provide a comprehensive B vitamin profile [18]. Hence, there is a need to develop a comprehensive method to measure each of the native forms of the B vitamins with a focus on mammalian tissues.

In this article we describe the development and validation of a new, quantitative method using hydrophilic interaction chromatography (HILIC) coupled to electrospray ionization tandem mass spectrometry for the simultaneous analysis of thirteen B vitamins and four 1C-related amines involved in Hcy remethylation. We apply the method to report, for the first time, a comprehensive profile of these compounds in the livers of 266 sheep.

## 2 Materials and methods

### 2.1 Materials

Solvents were all of HPLC grade. Ammonium formate, formic acid, acetic acid and acetonitrile were purchased from Fisher Scientific (Loughborough, UK). Unless otherwise stated, the following chemicals were purchased from Sigma-Aldrich (Poole, UK); 2-mercaptoethanol, citric acid; vitamin B12 standards: cyanocobalamin (CNCbl), coenzyme B_12_; adenosylcobalamin (AdoCbl), methylcobalamine hydrochloride (MeCbl); vitamin B6 standards: pyridoxine hydrochloride (PN), pyridoxamine dihydrochloride (PM), pyridoxal hydrochloride (PL), pyridoxal 5′-phosphate (PLP), 4-pyridoxic acid (PA); vitamin B2 standards: (-)-riboflavin (RF), riboflavin 5′-monophosphate sodium salt hydrate (FMN), flavin adenine dinucleotide disodium salt hydrate (FAD); vitamin B1 standards: thiamine hydrochloride (B1), thiamine pyrophosphate (TPP); amine standards: L-cystathionine (Cth), trimethylgycine (TMG), N,N-dimethylglycine (DMG) and methylglycine (MG). Deuterated internal standards (IS); pyridoxine-d4 (PND) and thiamine-d3 pyrophosphate chloride (TPPD), were purchased from Toronto Research Chemicals Inc. (Toronto, Canada). High purity water was produced by a Millipore water purification system (Millipore S.A.S., Molsheim, France).

### 2.2 Standard preparation and calibration

Stock solutions of B12, B6, B2, B1 and 1C amines were prepared in 0.01 M aqueous HCl. Methylcobalamin and two deuterated IS were prepared in pure methanol and all stock solutions were stored at −80 °C. Aqueous bovine serum albumin (10% BSA) was used as a surrogate matrix for the preparation of extracted calibration standards and these were prepared by adding 150 μL of extraction buffer (95% acetonitrile, 1% acetic acid, 0.1% ascorbic acid and 0.1% 2-mercaptoethanol) containing a suitable range of concentrations of standards into 50 μL of 10% BSA containing the IS mix.

### 2.3 Animals and experimental design

Fresh liver samples were collected immediately after slaughter from around 260 purebred weaned and pubertal Texel lambs which originated from five farms in the UK. Approximately half of these lambs were male. Diced sections (5 mm^3^) from the same region of the right lobe of the liver were collected on each occasion and snap frozen in liquid N_2_ within 15 min of exsanguination. These samples were stored at −80 °C until analyses. Although experimental procedures were not performed on these animals (liver samples were collected *post mortem*), the study was assessed by the Animal Welfare and Ethical Review Board at the University of Nottingham.

### 2.4 Sample preparation

50 mg of frozen ground liver were extracted with cold 300 μL of aqueous buffer (50% acetonitrile, 1% acetic acid, 0.1% ascorbic acid, 0.1% 2-mercaptoethanol and IS mix). Homogenization was performed using a Tissuelyser (RetschQiagene) with two disruption steps at 25 Hz for 2.5 min, freezing samples between steps. Liver homogenates were incubated at 50 °C in a water bath for 15 min, rapidly cooled on ice and centrifuged at 2000 *g* for 15 min. Further deproteination was undertaken by the addition of 100 μL of cold acetonitrile. The clear supernatant was transferred into an amber HPLC vial for LC-MS/MS analysis.

### 2.5 LC-MS/MS analysis

LC-MS/MS analyses were performed on a LC-10AD systems (Shimadzu, Kyoto, Japan) equipped with a SIL-HTC autosampler coupled to an ABI 4000 QTRAP tandem mass spectrometer using an electrospray ion source (Turbo Ion Spray™) (SCIEX, Foster City, CA) in positive ionisation mode. Chromatographic separation was performed on a Sequant ZIC–pHILIC column (150 × 4.6 mm, 5 μm particle size) with guard column (Sequant ZIC–pHILIC, 20 × 2.1 mm, 5 μm particle size) kept at 45 °C and with a flow rate of 0.4 mL/min. Mobile phases were composed of aqueous buffer solution (20 mM ammonium formate) adjusted to pH 3.5 for eluent A and 100% acetonitrile for eluent B. Gradient elution was carried out by the following program: isocratic hold 80% B for 1 min, next a linear gradient from 80% B to 5% B for 5 min, followed by a linear gradient back to 80% in 2 min, then isocratic hold on 80% for another 6 min. The total run time was 13 min. MS settings were as follows: ion source temperature (450 °C), ion spray voltage (5000 V), curtain gas (25 psig), collision gas (8 psig), ion source gas 1 (20 psig), ion source gas 2 (20 psig) interface heater activated. Analyst software (Applied Biosystems/MDS SCIEX) was used for HPLC system control and data acquisition and processing. The MS/MS acquisition method (MRM) was developed and the parameters are shown in Table 1. Two MRM transitions were monitored for each compound to use the ratio of quantifier and qualifier transition for compound identification. All compounds were quantified by the method of standard addition using an 8 point calibration constructed by plotting the ratio of the peak area of analyte to that of the relevant internal standard. TPPD was used for all phosphorylated compounds and PND was used for all non-phosphorylated compounds. For batch analysis bulk quality control (QC) samples were made from homogenized sheep liver supplemented with analyte standards. These QC samples were used to monitor assay performance across many batches.

**Table 1:** Mass spectrometer parameters for identification of B vitamins and 1C-related amines.

Analyte	Retention time (min)	Q1 Mass (amu)	Q3 Mass (amu)	DP	CE	CXP
Cyanocobalamin (CNCbl)	5.82	678.54	147.3	81	63	12
		678.54	359.0	81	35	12
Adenosylcobalamin (AdoCbl)	6.23	790.56	665.5	96	31	16
		790.56	147.2	96	73	10
Methylcobalamin (MeCbl)	5.9	673.16	147.2	76	77	12
		673.16	359.1	76	37	10
Pyridoxine (PN)	6.74	170.08	152.0	41	21	10
		170.83	134.2	41	31	10
Pyridoxamine (PM)	6.43	168.23	150.2	31	37	6
		168.23	94.2	31	37	6
Pyridoxal (PL)	8.26	169.21	134.1	56	31	1
Pyridoxal phosphate (PLP)	7.6	248.06	152.0	31	31	12
		248.06	134.4	31	41	10
Pyridoxic acid (PA)	5.52	184.21	148.1	56	31	10
		184.21	65.0	56	49	10
Riboflavin (RF)	5.83	377.27	243.1	106	35	20
		377.27	172.1	106	53	12
Flavin mononucleotide (FMN)	6.07	457.10	359.1	96	33	12
		457.10	198.1	96	73	14
Flavin adenine dinucleotide (FAD)	6.29	786.27	348.0	111	33	10
		786.27	136.0	111	61	8
Thiamine (B1)	7.11	265.15	122.1	26	21	8
		265.15	144.0	26	19	10
Thiamine pyrophosphate (TPP)	6.82	426.10	122.2	96	27	8
		426.10	81.2	96	47	8
Cystathionine (Cth)	7.35	223.11	134.1	56	21	6
		223.11	87.9	56	39	2
Trimethylglycine (TMG)	6.2	118.09	58.0	66	43	8
		118.09	59.1	66	27	10
Dimethylglycine (DMG)	6.41	104.03	58.2	23	21	2
		104.03	42.1	23	13	2
Methylglycine (MG)	6.91	89.87	44.1	36	19	6
		89.87	42.0	96	27	8
Pyridoxine-d4 (PND)	8.24	174.09	138.0	21	33	8
Thiamine-d3 pyrophosphate (TPPD)	6.82	428.20	125.0	96	61	12

### 2.6 Method validation

Method validation followed standard guidelines for bioanalytical method development [19]. 10% BSA in water was used as a surrogate blank matrix for validation experiments since this gave an equivalent protein load compared with sheep liver. The precision of method (RSD%) was determined by analysis of intra-day (n = 3) and inter-day samples (3 days, n = 9) under the same experimental conditions. Recovery was determined by spiking varying concentrations of vitamers into 6 liver homogenates at low (2.5 nM), medium (25 nM), and high (100 nM) concentrations. The matrix effect was evaluated by comparing the response of vitamins in standard solutions with the response of those in samples spiked with varying concentration of mixed standards after the deproteinisation step. Lower limit of detection (LOD, S/N = 3) and quantification (LOQ, S/N = 10) were determined in six replicates.

### 2.7 Statistical analysis

Data analysis was performed using Analyst software (Applied Biosystems/MDS, analytical Technologies, Concord, Ontario, Canada), version 1.4. The data processing included assessment of intra/inter-day precision of the assay and graphs were performed using Origin 8.1 software (OriginLab Corp).

The relationship between hepatic elemental cobalt (mg/kg fresh weight) and B12 vitamers (pmol/g fresh weight) was established by multiple linear regression using Genstat 17.1 (VSN International Ltd, Oxford, UK). Data were log_10_ transformed prior to analysis in order to normalize variance. Outputs generated were adjusted for farm of origin. The test subjects were pre-pubertal lambs slaughtered outside their natural breeding season (October to April). Therefore, there were no differences in liver metabolism between the two sexes, and so this term was dropped from the final statistical model.

## 3 Results and discussion

### 3.1 Optimisation of chromatography and mass spectrometry

B vitamins exhibit great structural diversity which confers a wide range of polarities and hence a wide range of chromatographic retention. Vitamin B1 and B6 species were highly hydrophilic while B2 vitamers were hydrophobic, resulting in difficulties for simultaneous measurement of water-soluble vitamins using conventional reversed-phase HPLC. Initially two different modes of chromatography were evaluated, reversed-phase ion-pair (RPIP) chromatography and hydrophilic interaction liquid chromatography (HILIC). Comparison of peak shape, retention time and response (data not shown) demonstrated that HILIC gave much improved chromatographic performance for the separation of these compounds which were eluted between 5 and 9 min with a total run time of 13 min (Fig. S1 in Supplementary Information).

All analytes exhibited a strong signal from the single protonated molecule [M+H]+ (Fig. S2 in Supplementary Information). Fragmentation of B1 occurs between two rings producing two product ions at *m*/*z* 144 (thiazolic ring) and 122 (pyrimidinic ring). Fragmentation of TPP also produced an abundant product ion at *m*/*z* 122 additional to product ions at *m*/*z* 304 and 126 generated by continuous loss of phosphate from the thiazolic ring.

Fragmentation of B2 gave a strong signal at *m*/*z* 243 following loss of a ribitol moiety and it further cleaved at the flavin ring to give two product ions at *m*/*z* 172 and 198. The fragmentation pattern of FMN was similar to B2 following the loss of the phosphate group while the major product ion for FAD was the protonated adenylic acid at *m*/*z* 349 [20]. All B6 non-phosphorylated forms shared a similar decomposition pattern following the loss of water, ammonia and phosphoric acid. Dissociation of PN and PA produced abundant ions at *m*/*z* 134 and 148, respectively, following loss of two H_2_O. The major product ion for PM, at *m*/*z* 150, was generated following loss of H_2_O while loss of ammonia and H_2_O was observed for PL to produce fragments at *m*/*z* 152 and 134.

Fragmentation of B12 vitamers; CNCbl, AdoCbl and MeCbl produced predominantly doubly charged ions. As ionization patterns of all vitamins were consistent with previous reports [12], [16], [21], [22], [M+2H]^2^+ for B12, [M]+ for B1 and [M+H]+ for all the other vitamins were selected as precursor ions for further MS/MS experiments.

### 3.2 Optimisation of sample extraction

The main purpose for this optimization was to maximize the liberation of vitamers from the matrix (sheep liver) with the minimum loss of analyte. Since some of the B vitamins are unstable and sensitive to degradation [6], a simple and short sample extraction was preferable to maximise the stability and recovery of analytes. Acetonitrile (5%) containing 1% acetic acid extraction solvent with 10 kDa molecular weight cut-off filters gave reasonable recoveries but extreme peak tailing was observed on pHILIC column (data not shown) due to the high percentage of water. The best combination of analyte extraction efficiency and peak shape was achieved using 50% acetonitrile containing 1% acetic acid for homogenization of liver sample with extraction at 50 °C for 15 min. A further protein precipitation step was included by addition of an equal volume of pure acetonitrile to the extract to obtain a clear supernatant for analysis. Our optimized extraction method agrees with previous reports that acid hydrolysis combined with heat treatment gives a high and reproducible recovery of B vitamins in a range of matrices [14], [17], [23], [24].

### 3.3 Method validation

Measured recoveries, matrix effect, accuracy and precision data for all analytes are shown in Table 2, which contains comprehensive validation information. Linearity for the standard calibration curves in 10% BSA were demonstrated by a correlation coefficient of ≥0.99 with typical linear ranges of 2 to 200 nmol/g covering the known ranges of endogenous concentrations of the B vitamins in liver. Generally, for all vitamins analysed the RSD% values for intra- and inter-day precision at low, medium and high concentrations were <15%, except for RF (17.4%), FMN (19.5%) and MG (17.2%). Ion enhancement was observed for B1 (261–422%) while ion suppression was observed for MeCbl (63–107%) and FMN (82–107%). However, these matrix effects can be corrected by using an isotope labeled internal standard (e.g. TPPD or PND). Overall, this method was suitable for quantification of B vitamins and related compounds in a protein-based matrix.

**Table 2:** Validation data for recovery of B vitamins and 1C-related amines in sheep liver.

Analytes	Recovery (%)			Matrix effect (%)			Accuracy (%)			Intra-day Precision (%)			Inter-day Precision (%)			LOD (nmol/g)	Linear range (nmol/L)*	Slope	R^2^
	L	M	H	L	M	H	L	M	H	L	M	H	L	M	H				
**CNCbl**	71	79	85	125	82	82	94	106	103	5.5	6.5	10.3	9.9	11.0	6.2	2.0	0.4–50	0.6126	0.9967
**AdoCbl**	73	83	89	136	111	91	92	96	104	9.2	3.1	6.7	8.8	5.2	5.6	2.0	0.4–50	0.097	0.9937
**MeCbl**	60	87	102	74	63	107	94	81	68	13.2	9.3	10.2	7.4	11.1	9.7	1.5	0.8–100	0.0753	0.9951
**PN**	100	76	81	95	117	98	75	82	90	5.6	1.6	6.1	6.8	9.4	6.7	1.6	0.8–100	0.1505	0.9976
**PM**	105	75	73	75	110	86	77	82	89	10.6	3.0	7.4	5.7	9.6	7.7	0.4	0.8–100	0.2591	0.9988
**PL**	60	69	90	113	94	90	88	93	105	13.4	9.0	3.4	8.3	14.3	11.9	1.6	0.8–100	0.2801	0.9997
**PLP**	119	111	102	67	111	105	91	99	98	12.5	9.2	13.4	3.6	11.6	10.5	0.4	0.4–50	0.2801	0.9945
**PA**	86	87	90	97	110	86	99	107	106	6.0	3.0	2.3	13.6	14.7	7.9	1.6	0.4–50	0.9844	0.9912
**RF**	76	71	56	109	103	104	73	91	94	7.8	2.4	4.1	12.2	17.4	12.1	1.2	4–500	0.3045	0.9927
**FMN**	79	84	85	82	107	86	108	92	98	5.4	7.5	2.4	11.3	19.5	12.5	1.6	2.3–300	0.0354	0.9990
**FAD**	67	101	98	79	104	87	102	103	97	5.4	4.5	5.9	9.3	7.3	8.5	24.0	8–10000	0.01086	0.9889
**B1**	102	105	104	261	402	422	97	96	99	6.7	2.8	5.3	12.8	10.2	9.7	0.5	2.34–300	0.8777	0.9991
**TPP**	43	85	105	85	84	67	91	99	98	12.5	9.2	13.4	5.8	8.2	6.7	2.0	2.34–300	0.1369	0.9939
**Cys**	92	91	94	102	107	88	93	95	101	6.4	2.1	7.1	13.2	10.7	11.3	2.0	4–500	0.0006	0.9974
**TMG**	108	97	114	91	99	93	102	121	100	3.4	4.8	3.4	9.3	7.1	10.3	3.2	4–500	0.0011	0.9934
**DMG**	83	72	92	106	99	91	93	123	96	8.0	6.6	13.0	12.4	11.3	9.1	0.5	2.3–300	0.0080	0.9945
**MG**	122	83	93	129	134	103	156	107	93	2.5	1.5	4.1	16.1.	17.2	10.2	1.8	4–500	0.0039	0.9943

* nmol/ml LOD (derived from standards spiked in 10% BSA) is equivalent to nmol/g in fresh liver tissue.

### 3.4 Determination of B vitamins and related amines in sheep liver

**Fig. 2:**
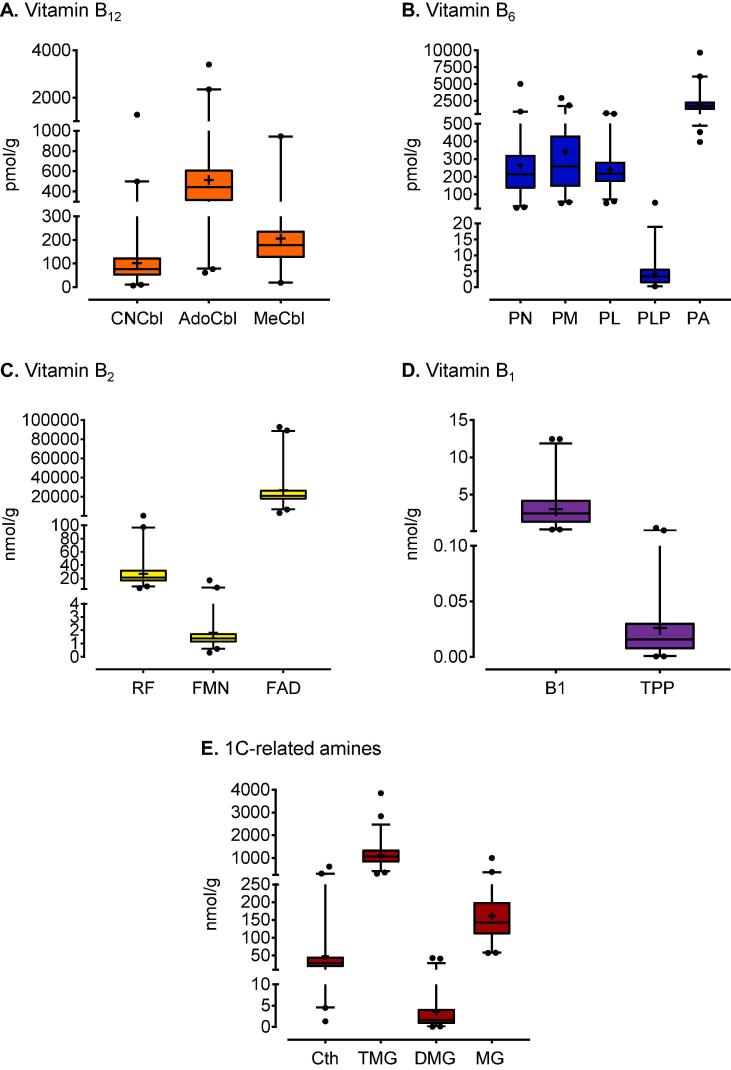
Concentrations of B vitamins in sheep liver. CNCbl, cyanocobalamin; AdoCbl, adenosylcobalamin; MeCbl, methylcobalamin (**A**). PM, pyridoxamine; PN, pyridoxine; PL, pyridoxal; PLP, pyridoxal 5′-phosphate; PA, pyridoxic acid (**B**). RF, riboflavin; FMN, flavin mononucleotide; FAD, flavin adenine dinucleotide (**C**). B1, thiamine; TPP, thiamine pyrophosphate (**D**). Cth, cystathionine; TMG, trimethylglycine; DMG, dimethylglycine; MG, methylglycine (**E**). Boxplots depict mean (+), median and interquartile ranges with whiskers set at 1st and 99th percentiles.

The validated method enabled us, for the first time, to measure accurately the levels of B vitamins and specific 1C amines related to Hcy metabolism in sheep liver (Fig. 1) from a single sample without the need for multiple methods. We have used the sheep study to exemplify how 1C intermediates can be profiled in solid tissues or biological fluids to gain further insights into the role of B vitamins in tissue-specific 1C cellular metabolism. We analysed 266 liver samples from a single genotype of sheep (Texel) originating from five farms representing different geographical regions of England. A summary of B vitamin and related 1C amine profiles obtained from these samples is presented in Fig. 2.

We observed that thiamine (B1) was the predominant form in sheep liver while the bioactive form, TPP [25], accounted for <5% of total B1 concentrations. B1 deficiency is common in weaned lambs of the type that partook in this study, which may partly explain why B1 concentrations in the livers of our test animals were low relative to values reported more generally in sheep (Table S2 in Supplementary Information). Diets containing high levels of fermentable-starch or sulphur, and/or ingestion of moldy feed containing high levels of thiaminases can further increase the incidence and severity of B1 deficiency in sheep leading to the commonly observed condition of polioencephalomalacia [26], [27].

Flavin adenine dinucleotide was the major form of B2 measuring almost ~ 40-fold higher than its precursors, RF and FMN. As for B1, relatively low levels of hepatic B2 in our test animals (Table S2 in Supplementary Information) may reflect their relatively immature age and stage of rumen development [28], [29]. Among the five measured B6 species, the catabolite of B6, pyridoxic acid (PA) [30], was the predominant form, followed by dietary precursors, PM and PN. Only trace levels of the bioactive form of B6, PLP, were detected in the liver using our method. The level of AdoCbl in sheep liver was >2-fold higher than MeCbl. These vitamers are cofactors of methylmalonyl-CoA mutase (MUT) and methionine synthase (MTR), respectively [31] (Fig. 1).

The most abundant 1C amines measured in sheep liver were trimethylglycine (betaine) and methylglycine (sarcosine). Trimethylglycine (TMG) donates a methyl group to convert Hcy to methionine, thereby generating dimethylglycine (DMG) which is subsequently metabolized to methylglycine (MG) [32]. Cystathionine (Cth) is the first metabolic intermediate produced by condensation of serine with Hcy in the vitamin B6-dependent two-step transsulphuration pathway [33], [34] (Fig. 1).

As seen from Fig. 2, there are large natural variations in the levels of individual vitamers and amines across the sheep population. Concentrations of B vitamins in animal tissues have been reported previously (Table S2 in Supplementary Information). However, as total concentrations were measured, no knowledge can be gained about the variation and tissue-specific distribution of bioactive vitamers. The measurement of specific coenzyme forms will facilitate more accurate deficiency diagnosis.

The total concentrations of B vitamins reported herein coincide with those reported previously except for vitamin B6, where concentrations reported in literature are >10-fold higher. This discrepancy may be due to differences in analytical methodologies employed. Previous studies converted all B6 vitamers, including their phosphate esters, into free forms (i.e. PN, PM and PL) [35], [36] before measuring total B6 by microbiological assay [35] or reversed-phase HPLC coupled with electrochemical detection [36], [37], [38]. Such approaches are quantitative but they do not discriminate between different free and phosphorylated vitamers reviewed by [39]. In the present study we elected to measure only free forms of B6, to gain an insight into their relative abundance, together with the bioactive form, PLP, as free forms are phosphorylated upon cellular uptake (Fig. 1) [40].

### 3.5 Clinical interpretation: Relationship between liver cobalt and cobalamin

**Fig. 3:**
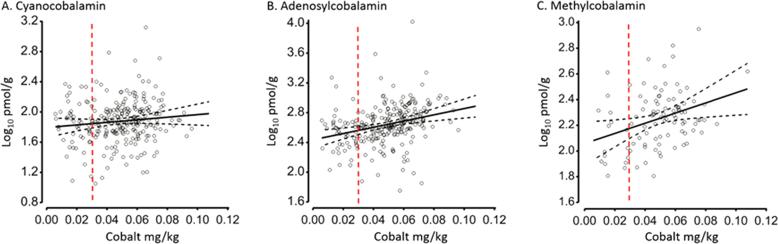
Relationship between elemental cobalt (mg/kg fresh weight) and B12 vitamers (log_10_ pmol/g fresh weight) in sheep liver. Outputs generated using multiple regression models adjusted for farm of origin. In contrast to cyanocobalamin (**A**), liver concentrations of which were not related to cobalt, both liver adenosylcobalamin (**B**) and methylcobalamin (**C**) increased (P < 0.001) with cobalt. Red dotted lines represent the lower marginal threshold for liver cobalt concentrations in sheep; below which animals, over time, exhibit signs of clinical B12 deficiency [42]. Black dotted lines represent 95% confidence intervals.

Ruminal synthesis of B12 from elemental cobalt is adequate to meet the metabolic requirements of ruminants including sheep under most circumstances provided dietary sources of this element are non-limiting [41], [42]. Herbage availability of cobalt is determined by underlying regional geology, soil series and species of plant [43], [44], and is correlated with ruminal concentrations of B12 in sheep [45]. In the current study, sheep were sampled from 5 geographical regions of England and Wales and approximately 20% were identified as having liver cobalt levels (see Supplementary Information) below the recognised lower marginal threshold for this species (Fig. 3). Multiple regression analyses, with geographical region fitted to the model, revealed significant (P < 0.001) positive relationships between hepatic B12 and cobalt concentrations, but for the two coenzyme forms (i.e. adenosylcobalamin and methylcobalamin) only (Fig. 3). Whilst all three vitamers can be synthesized from cobalt by rumen microbes [46], in some cases cyanocobalamin may also have been incorporated as a supplement in the diet of our test animals, as this is common practice. Absorbed dietary cyanocobalamin therefore could in part explain its lack of association with hepatic cobalt concentrations. Hydroxycobalamin and additional analogues (e.g. cobamides and cobinamides) were not measured in the current study but are also found in sheep liver; the former constituting around 25% of corrinoids [47].

The abundance of adenosylcobalamin relative to methylcobalamin (>2-fold) is consistent with reports that B12 is preferably stored in this form in the liver [31], [48]. Attached to methylamalonylCoA mutase, high levels of adensylcobalamin reflect the importance of propionate metabolism (Fig. 1) in gluconeogenesis in sheep [47]. It is possible that the relative concentration of these vitamers in circulation will differ from that found in liver [47]. This will be determined in future experiments which will explore the effects of deficiencies in dietary cobalt in this and related ruminant species now that a sensitive method to facilitate quantification of these two coenzyme forms of B12 has been developed.

## 4 Conclusions

The validated method described herein is capable of the simultaneous quantification of 13 B vitamins and four related compounds in a complex tissue matrix (i.e. sheep liver). It uses a simple sample extraction procedure followed by a short run time and a simple mobile phase. We demonstrate that it also provides good sensitivity and reliability for the simultaneous determination of B vitamins and associated 1C amines in sheep liver. Such analyses will be invaluable for modelling one-carbon and linked propionate metabolic pathways in order to facilitate studies that seek to investigate both dietary and genetic contributions to metabolic health and epigenetic gene regulation.

## Declaration of Competing Interest

The authors declare that they have no known competing financial interests or personal relationships that could have appeared to influence the work reported in this paper.
