# Supplementary material for: Comprehensive and quantitative profiling of B vitamins and related compounds in the mammalian liver
Source: J Chromatogr B Analyt Technol Biomed Life Sci. 2020 Jan 1;1136:121884. doi: 10.1016/j.jchromb.2019.121884 (PMC6961113; doi:10.1016/j.jchromb.2019.121884)
Supplement: Supplementary data 1 [file mmc1.pdf]

Appendix A. Supplementary Information

ICP-MS analysis

Frozen liver samples (1 g) were freeze dried. Approximately 0.1 g of freeze dried liver was wet-digested in 3 mL 70% HNO_3, 2 mL 30% H_2O_2 (Fisher Scientific, UK) and 3 mL Milli-Q water (18.2 MΩ cm) for 45 minutes in a Multi-wave PRO microwave (Anton Paar, UK). Digested samples were washed from digestion vessels into universal tubes (Sarstedt, Leicester, UK) with sequential aliquots (3 mL and 4 mL) of Milli-Q water. Blank samples and certified reference material (1577c bovine liver, National Institute of Standards and Technology, US) were included in each acid digest batch.

The elemental cobalt concentration of liver digests was determined using inductively coupled plasma mass spectrometry (ICP-MS) (iCAP-Q; Thermo-Fisher Scientific, Bremen, Germany). The instrument was operated in collision-cell mode (Q cell) using He with kinetic energy discrimination to remove polyatomic interferences (He-cell). Samples were introduced using an autosampler (Cetac ASX-520) incorporating an ASXpress™ rapid uptake module through a PEEK nebulizer (Burgener Mira Mist). Internal standards, Ge (10 µg/L), Rh (110 µg/L) and Ir (5 µg/L) in 2% trace analysis grade (Fisher Scientific, UK) HNO_3, were introduced to the sample stream on a separate line via the ASXpress unit. Cobalt calibration standards (Claritas-PPT grade CLMS-2 from SPEX Certiprep Inc., Metuchen, NJ, USA) were in the concentration range of 0 – 100 µg/L. Sample processing was conducted using Qtegra™ software (Thermo-Fisher Scientific) utilizing external cross-calibration between pulse-counting and analogue detector modes.

Figure Legends (Appendix A. Supplementary Information)

Figure S1. LC-MS/MS chromatograms of B vitamins in sheep liver (**A**). LC-MS/MS chromatograms of B vitamins in sheep liver spiked with standards (**B**). Abbreviations: methylcobalamin, MeCbl; adenosylcobalamin, AdoCbl; cyanocobalamin, CNCbl; pyridoxal 5'-phosphate, PLP; pyridoxic acid, PA; pyridoxine, PN; pyridoxamine, PM; pyridoxal, PL; flavin adenine dinucleotide (FAD); flavin mononucleotide, FMN; riboflavin, RF; thiamine pyrophosphate (TPP); thiamine, B1.

Figure S2. Mass spectra of B vitamins: cyanocobalamin, CNCbl (**A**); adenosylcobalamin, AdoCbl (**B**); methylcobalamin, MeCbl (**C**); pyridoxine, PN (**D**); pyridoxamine, PM (**E**); pyridoxal, PL (**F**); pyridoxal 5'-phosphate, PLP (**G**); riboflavin, RF (**H**); flavin mononucleotide, FMN (**I**); flavin adenine dinucleotide, FAD (**J**); thiamine, B1 (**K**); thiamine pyrophosphate, TPP (**L**). Abbreviations: Rel. Int, relative intensity %; m/z, mass/charge; amu, atomic mass units; CE, collision energy; MCA, multi-channel analysis; cps, counts per second.

Figures (Supplementary)

A

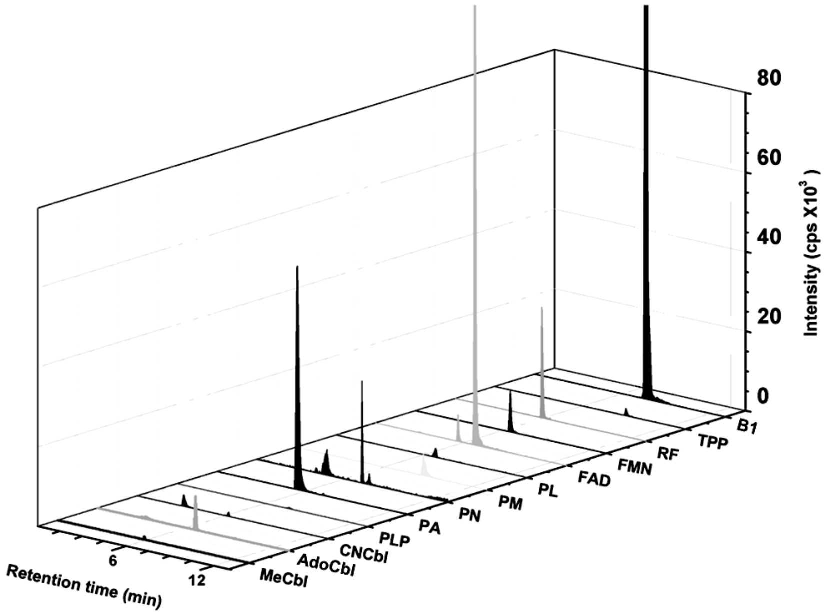

B

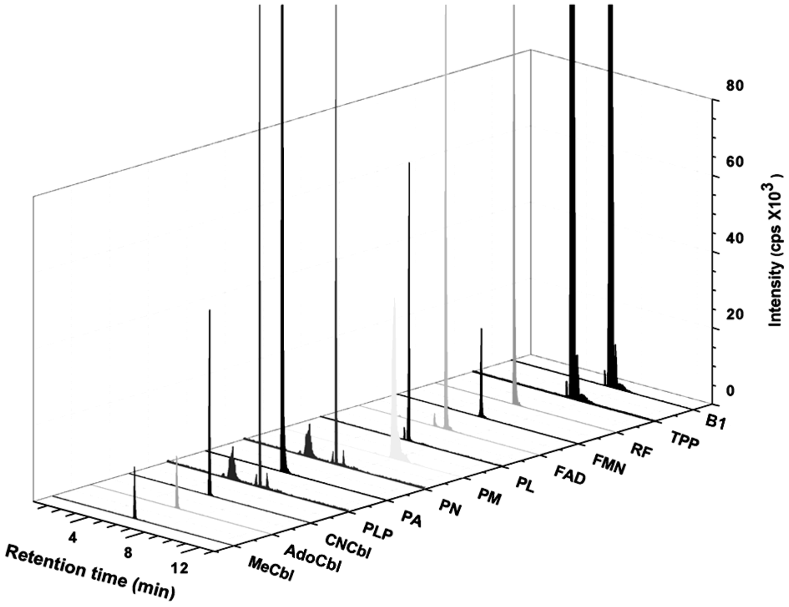

Figure S1.

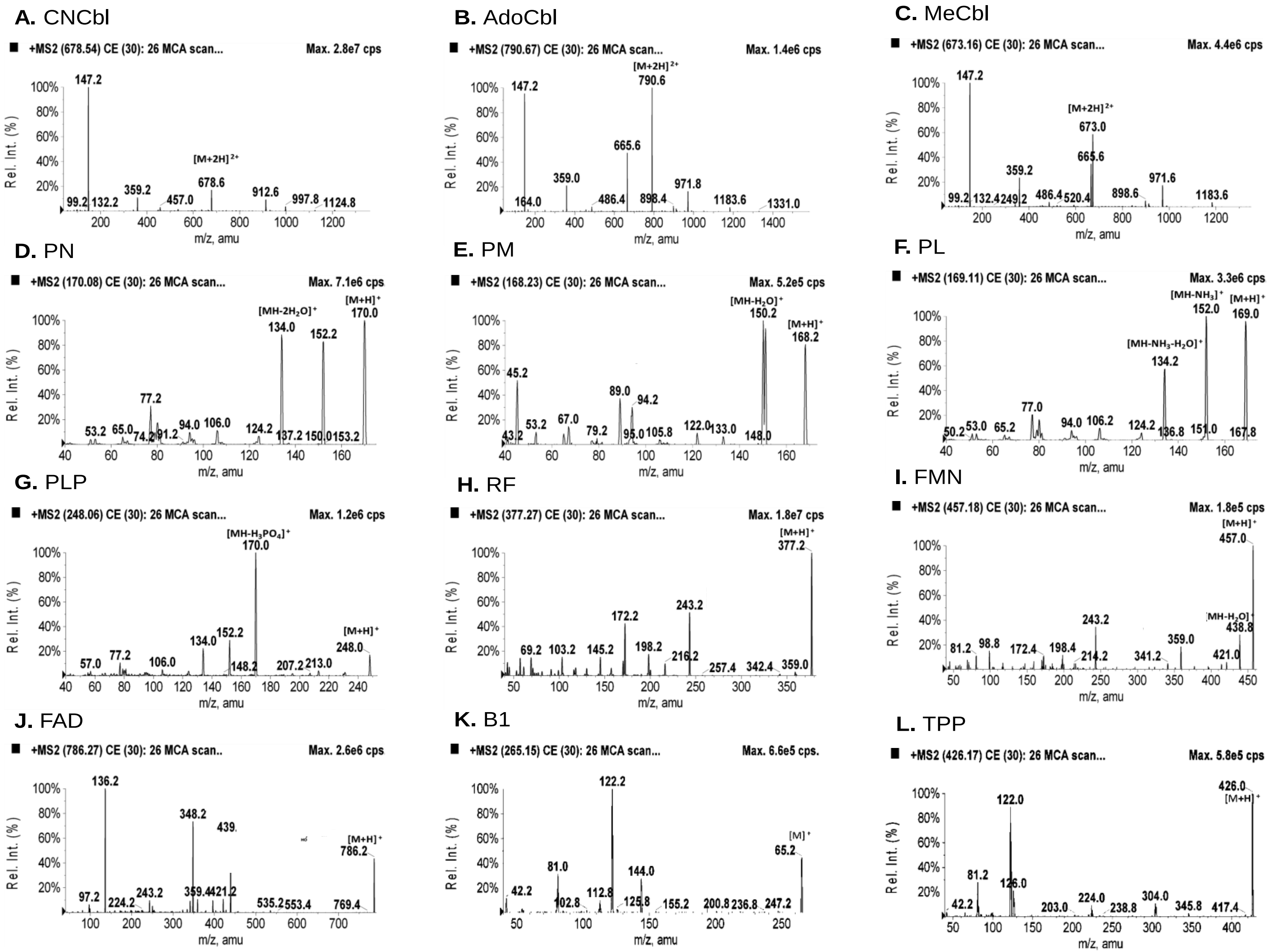

A. CNCbl
+MS2 (678.54) CE (30): 26 MCA scan...
Max. 2.8e7 cps
B. AdoCbl
+MS2 (790.67) CE (30): 26 MCA scan...
Max. 1.4e6 cps
C. MeCbl
+MS2 (673.16) CE (30): 26 MCA scan...
Max. 4.4e6 cps
D. PN
+MS2 (170.08) CE (30): 26 MCA scan...
Max. 7.1e6 cps
E. PM
+MS2 (168.23) CE (30): 26 MCA scan...
Max. 5.2e5 cps
F. PL
+MS2 (169.11) CE (30): 26 MCA scan...
Max. 3.3e6 cps
G. PLP
+MS2 (248.06) CE (30): 26 MCA scan...
Max. 1.2e6 cps
H. RF
+MS2 (377.27) CE (30): 26 MCA scan...
Max. 1.8e7 cps
I. FMN
+MS2 (457.18) CE (30): 26 MCA scan...
Max. 1.8e5 cps
J. FAD
+MS2 (786.27) CE (30): 26 MCA scan..
Max. 2.6e6 cps
K. B1
+MS2 (265.15) CE (30): 26 MCA scan...
Max. 6.6e5 cps.
L. TPP
+MS2 (426.17) CE (30): 26 MCA scan...
Max. 5.8e5 cps
Rel. Int. (%)
m/z, amu

Figure S2.

Tables (Appendix A. Supplementary Information)

Table S1. Structure and function of B vitamin cofactors involved in 1C metabolism and related pathways.

CNCbl	**Cyanocobalamin (B12)**	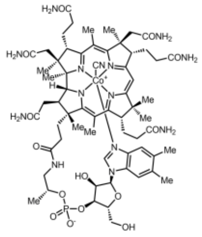	Synthetic form of vitamin B_{12} that is biologically inert but converted to bioactive cofactors; AdoCbl and MeCbl [1, 2]	PN	**Pyridoxine (B6)**	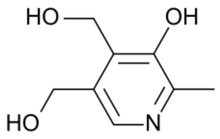	Alcohol form of vitamin B_6 that is commonly found in food and dietary that is readily converted to bioactive forms [3]
AdoCbl	**Adenosylcobalamin (B12)**	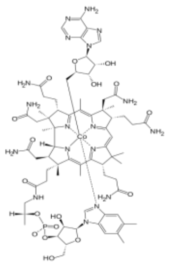	Cofactor for mitochondrial MUT enzyme that catalyses isomerisation of methylmalonyl-CoA to succinyl-CoA in propionate metabolism [4]	PM	**Pyridoxamine (B6)**	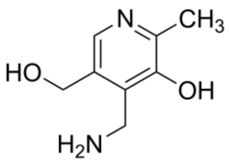	Amine form of vitamin B_6 that is readily transformed to PL [5]
MeCbl	**Methylcobalamin (B12)**	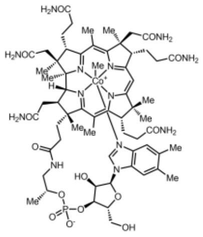	Cofactor for MTR enzyme that catalyses remethylation of Hcy to Met in methionine cycle [4]	PL	**Pyridoxal (B6)**	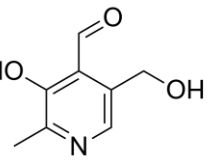	Aldehyde form of vitamin B_6 that is phosphorylated to form the bioactive cofactor, PLP [3]

Table S1 continued. Structure and function of B vitamin cofactors involved in 1C metabolism related pathways.

Cofactor	Name	Structure	Function
**RF**	**Riboflavin (B2)**	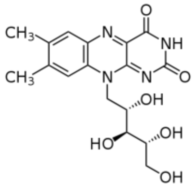	Dietary form of vitamin B_2 that serves as a precursor for formation of bioactive forms. RF is phosphorylated to FMN [6]
**PLP**	**Pyridoxal 5' phosphate (B6)**	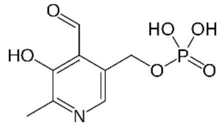	Cofactor for SHMT enzyme that catalyses simultaneous conversion of Ser to Gly, and THF to CH_2THF in the folate cycle [7]<br>Cofactor for CBS and CTH enzymes that catalyse the conversion of Hcy first to Cth and then to Cys in the transsulphuration pathway [8]
**FMN**	**Flavin mononucleotide (B2)**	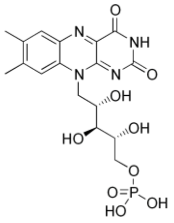	Cofactor for MTRR enzyme that restores MTR activity for remethylation of Hcy to Met in methionine cycle [9]
**B_1**	**Thiamine (B1)**	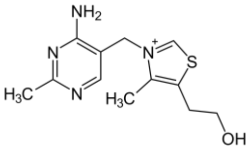	Form of vitamin B_1 that is rapidly phosphorylated to bioactive form, TPP [10]
**FAD**	**Flavin adenine dinucleotide (B2)**	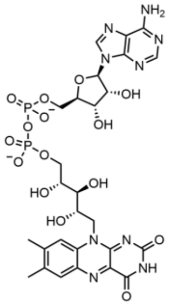	Cofactor for MTHFR enzyme that catalyses conversion of CH_2THF to 5-methyltetrahydrofolate 5-mTHF in the folate cycle<br>Cofactor for MTRR enzyme that restores MTR activity for remethylation of Hcy to Met in methionine cycle [9]
**TPP**	**Thiamine pyrophosphate (B1)**	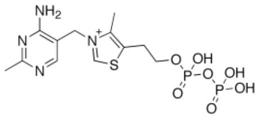	Cofactor for enzymes involved in oxidative phosphorylation; PDH catalyses the decarboxylation of pyruvate to acetyl-CoA and KGDH catalyses the conversion of α-KG to succinyl-CoA in the TCA cycle [11, 12]

Table S2. Total concentration ranges of B vitamins in animal tissues.

	Current study	Reported concentration range*		
**Vitamin B_{12}**	***Liver* (pmol/g)**	***Liver* (pmol/g)**	***Muscle* (pmol/g)**	***Blood* (pmol/L)**
Sheep	713	96-881[13-16]	0.6-7[14,15,17]	164-2757[17-19]
Cow		342-1266[14,15,17]	3-8[14,15,17]	70-102[17]
Deer		428-1024[15,20]	20-21[15]	65-530[36]
Pig		149-183[15,22]	3-4[15]	50-127[22,23]
Rat		15-100[24-27]	8-11[27]	3000-7180[24,25]
Chicken		132-133[15]	2-3[15]	-
**Vitamin B_6**				
Sheep	2657	28937[14,15]	5371-43678[14,15]	-
Cow		40402[15]	8190-28390[15]	-
Deer		28937[15]	10374[15]	-
Pig		34942[15]	12558[15]	-
Rat		20000-48900[24,25]	-	1.0-2.1[24,25]
Chicken		43668[15]	20747[15]	-
**Vitamin B_2** (Riboflavin)	**(nmol/g)**	**(nmol/g)**	**(nmol/g)**	**(nmol/L)**
Sheep	27	74-93[14,15]	4-7[14,15]	-
Cow		74-128[14,15]	3-6[14,15]	-
Deer		69-70[15]	6-7[15]	-
Pig		75-76[15]	3-4[15]	-
Rat		70-100[24,25]	-	169[24,25]
Chicken		90-91[15]	4-5[15]	-
**Vitamin B_1**				
Sheep	3	9-15[14,15]	3-6[14,15]	67-227[28]
Cow		8-12[14,15]	1-4[14,15]	71-237[28]
Deer		12-13[15]	3-4[15]	-
Pig		16-17[15]	15-16[15]	-
Rat		30-40[24,25]	-	346-400[24,25]
Chicken		23-24[15]	5-6[15]	-

Bibliography (Appendix A. Supplementary Information)

[1] Farquharson J and Adams JF (1976) The forms of vitamin B12 in foods. *Br J Nutr.* 36(1): 127-36

[2] Martinelli D, Deodato F, Dionisi-Vici C (2011) Cobalamin C defect: natural history, pathophysiology, and treatment. *J Inherit Metab Dis.* 34(1): 127-35

[3] Albersen M, Bosma M, Knoers NV, de Ruiter BH, Diekman EF, *et al*. (2013) The intestine plays a substantial role in human vitamin B6 metabolism: a Caco-2 cell model. *PLoS One.* 8(1): e54113

[4] Shane B (2008) Folate and vitamin B12 metabolism: overview and interaction with riboflavin, vitamin B6, and polymorphisms. *Food Nutr Bull.* 29(2 Suppl): S5-16

[5] Sakurai T, Asakura T, Mizuno A, Matsuda M (1992) Absorption and metabolism of pyridoxamine in mice. II. Transformation of pyridoxamine to pyridoxal in intestinal tissues. *J Nutr Sci Vitaminol (Tokyo).* 38(3): 227-33

[6] Powers HJ (2003) Riboflavin (vitamin B-2) and health. *Am J Clin Nutr.* 77(6): 1352-60

[7] Appaji Rao N, Ambili M, Jala VR, Subramanya HS, Savithri HS (2003) Structure-function relationship in serine hydroxymethyltransferase. *Biochim Biophys Acta.* 1647(1-2): 24-29

[8] Perry C, Yu S, Chen J, Matharu KS and Stover PJ (2007) Effect of vitamin B6 availability on serine hydroxymethyltransferase in MCF-7 cells. *Arch Biochem Biophys.* 432(1): 21-27
[9] García-Minguillán CJ, Fernandez-Ballart JD, Ceruelo S, Ríos L, Bueno O, *et al*. (2014) Riboflavin status modifies the effects of methylenetetrahydrofolate reductase (MTHFR) and methionine synthase reductase (MTRR) polymorphisms on homocysteine. *Genes Nutr.* 9(6): 435
[10] de Jong L, Meng Y, Dent J and Hekim S (2004) Thiamine Pyrophosphate Biosynthesis and Transport in the Nematode Caenorhabditis elegans. *Genetics*. 168(2): 845-54
[11] Lonsdale D (2015) Thiamine and magnesium deficiencies: keys to disease. *Med Hypotheses.* 84(2): 129-34
[12] McLain AL, Szweda PA and Szweda LI (2011) α-Ketoglutarate dehydrogenase: a mitochondrial redox sensor. *Free Radic Res.* 45(1): 29-36
[13] Indyk HE, Persson BS, Caselunghe MC, Moberg A, Filonzi EL, *et al*. (2002) Determination of vitamin B12 in milk products and selected foods by optical biosensor protein-binding assay: method comparison. *J AOAC Int.* 85(1): 72-81
[14] Williams PG (2007) Nutritional composition of red meat. *Nutrition and Dietetics.* 64(4): S113-19
[15] Hassan AA, Torkjel MS and Brustad M (2012) Level of selected nutrients in meat, liver, tallow and bone marrow from semi-domesticated reindeer (Rangifer t. tarandus L.). *Int J Circumpolar Health.* 71: 17997
[16] Kelly RJ, Gruner TM, Furlong JM and Sykes AR (2006) Analysis of corrinoids in ovine tissues. *Biomed Chromatogr.* 20(8): 806-14
[17] Ortigues-Marty I, Micol D, Prache S, Dozias D and Girard CL (2005) Nutritional value of meat: the influence of nutrition and physical activity on vitamin B12 concentrations in ruminant tissues. *Reprod Nutr Dev.* 45(4): 453-67
[18] Sinclair KD, Allegrucci C, Singh R, Gardner DS, Sebastian S, *et al*. (2007) DNA methylation, insulin resistance, and blood pressure in offspring determined by maternal periconceptional B vitamin and methionine status. *Proc Natl Acad Sci U S A.* 104(49): 19351-6
[19] Mitchell LM, Robinson JJ, Watt RG, McEvoy TG, Ashworth CJ, *et al*. (2007) Effects of cobalt/vitamin B12 status in ewes on ovum development and lamb viability at birth. *Reprod Fertil Dev.* 19(4): 553-62
[20] Tremain-Boon SG, Hart JC, Wilson PR and Lopez-Villalobos N (2002) Liver copper, selenium and vitamin B12 concentrations in farmed and feral red deer (Cervus elaphus). *N Z Vet J.* 50(3): 111-4
[21] Audigé L, Wilson PR and Morris RS (1999) Reproductive performance of farmed red deer (Cervus elaphus) in New Zealand. IV. Biological markers as risk factors for yearling and adult hind conception. *Anim Reprod Sci.* 55(3-4): 239-54
[22] Matte JJ, Guay F, Le Floc'h N and Girard CL (2010) Bioavailability of dietary cyanocobalamin (vitamin B12) in growing pigs. *J Anim Sci.* 88(12): 3936-44
[23] Guay F, Jacques Matte J, Girard CL, Palin MF, Giguère A, *et al*. (2002) Effects of folic acid and vitamin B12 supplements on folate and homocysteine metabolism in pigs during early pregnancy. *Br J Nutr.* 88(3): 253-63
[24] Fukuwatari T, Wada H and Shibata K (2008) Age-related alterations of B-group vitamin contents in urine, blood and liver from rats. *J Nutr Sci Vitaminol (Tokyo).* 54(5): 357-62
[25] Shibata K, Shimizu A and Fukuwatari T (2013) Vitamin B1 Deficiency Does not Affect the Liver Concentrations of the Other Seven Kinds of B-Group Vitamins in Rats. *Nutr Metab Insights.* 6: 1–10
[26] Birn H, Nexø E, Christensen EI and Nielsen R (2003) Diversity in rat tissue accumulation of vitamin B12 supports a distinct role for the kidney in vitamin B12 homeostasis. *Nephrol Dial Transplant.* 18(6): 1095-100
[27] Kornerup LS, Fedosov SN, Juul CB, Greibe E, Heegaard CW2, *et al*. (2018) Tissue

distribution of oral vitamin B12 is influenced by B12 status and B12 form: an experimental study in rats. *Eur J Nutr.* 57(4): 1459-69
[28] Hill JH, Rammell CG and Forbes S (1988) Blood thiamine levels in normal cattle and sheep at pasture. *N Z Vet J.* 36(2): 49-50
